# Anti-TIF-1γ Antibody Detection Using a Commercial Kit vs In-House Immunoblot: Usefulness in Clinical Practice

**DOI:** 10.3389/fimmu.2020.625896

**Published:** 2021-02-01

**Authors:** Anaís Mariscal, Milena Milán, Andrés Baucells, Maria Angeles Martínez, Andrea Garcia Guillen, Ernesto Trallero-Araguás, Marcelo Alvarado-Cardenas, Laura Martínez-Martínez, Leticia Alserawan, Teresa Franco-Leyva, María Teresa Sanz-Martínez, Laura Viñas-Giménez, Hector Corominas, Cándido Juárez, Iván Castellví, Albert Selva-O’Callaghan

**Affiliations:** ^1^Immunology Department, Hospital de la Santa Creu i Sant Pau, Biomedical Research Institute Sant Pau (IIB Sant Pau), Barcelona, Spain; ^2^Rheumatology Department, Hospital de la Santa Creu i Sant Pau, Biomedical Research Institute Sant Pau (IIB Sant Pau), Barcelona, Spain; ^3^Rheumatology Unit, Hospital Vall d’Hebrón, Barcelona, Spain; ^4^Internal Medicine Department, Hospital Vall d’Hebrón, Barcelona, Spain; ^5^Universitat Autónoma de Barcelona, Barcelona, Spain; ^6^Immunology Department, Hospital Vall d’Hebrón, Barcelona, Spain

**Keywords:** dermatomyositis, cancer, autoantibody, diagnosis, immunoassay

## Abstract

**Objectives:**

Anti-TIF-1γ autoantibody detection is important for cancer screening in patients with dermatomyositis. The gold standard for anti-TIF-1γ detection, immunoprecipitation, is only available from a few specialized laboratories worldwide, so commercial ELISA/immunoblot tests have emerged in recent years. To analyze their usefulness in diagnosing cancer-associated dermatomyositis, we compared Euroimmun Euroline profile with our previously validated in-house immunoblot assay with human recombinant TIF-1γ.

**Methods:**

We included 308 adult patients from Hospital de la Santa Creu I Sant Pau and Vall Hebrón Hospital (Barcelona, Spain) tested for anti-TIF-1γ autoantibodies using the Euroline profile and an in-house immunoblot assay.

**Results:**

A total of 27 anti-TIF-1γ were detected by the Euroline and 12 by the in-house assay. Fair agreement was observed between Euroline and the in-house immunoblot Cohen’s kappa 0.3163. Expected prevalence of anti-TIF-1γ autoantibodies was observed for the two methods for dermatomyositis and undifferentiated connective tissue diseases, but unexpectedly high prevalence of anti-TIF-1γ autoantibodies was detected by Euroline compared to the in-house immunoblot for other diseases (16.5% Euroline vs 0.8% in-house immunoblot, p<0.01). The in-house IB compared to Euroline more reliably detected cancer in patients with DM with anti-TIF-1γ antibodies (p=0.0014 vs p=0.0502 for in-house immunoblot vs Euroline).

**Conclusion:**

We recommend using a second validated method to confirm Euroline-detected anti-TIF-1γ antibodies when the dermatomyositis diagnosis is not definitive. Furthermore, in the context of definite DM diagnosis with negative anti-TIF-1γ antibodies by Euroline and no other myositis specific antibody, is also recommendable to confirm by a second validated method.

## Introduction

Anti-TIF-1γ autoantibodies (also anti-TRIM33 or anti-p155/140 autoantibodies) were first described in 2006 when a large cohort of patients with idiopathic inflammatory myopathies was examined by immunoprecipitation (IP) (1). In patients with dermatomyositis (DM), the prevalence of anti-TIF-1γ autoantibodies ranged from 14% (2) to 33% (1).

There is a strikingly increased risk of cancer in patients with myositis compared to the general population (3), with 32% of patients with DM reported to have an associated cancer diagnosis at some point during their illness (4). It is believed that around one third of DM cases are paraneoplastic (5). Anti-TIF-1γ autoantibodies are associated with DM and have been shown to be the best available biomarker for cancer-associated DM (CAM) (1, 6, 7). In fact, a patient with DM who is positive for anti-TIF-1γ autoantibodies has 27 times greater odds of having CAM compared to a patient with DM negative for anti-TIF-1γ (7).

Gold standard technique for anti-TIF-1γ autoantibody detection is the visualization of a 155 kDa band by K562 or Hela cells IP. However, the methodology is lengthy and complex and requires special facilities and trained staff for both technical and interpretation aspects. Protein-based assays—either ELISA or immunoblot (IB)—have the advantage, compared to IP, of unequivocal results specificity given that the substrate is a known protein; they therefore avoid the uncertainty associated with IP in identifying different proteins with similar molecular weights. In-house ELISA and IB assays with human recombinant TIF-1γ have been independently developed and validated by two groups (8, 9), while a commercial ELISA for anti-TIF-1γ developed by Medical and Biological Laboratories (MBL; Nagoya, Japan) has been validated against IP (10). Three commercial IB assays are also available for myositis-specific antibody detection, including anti-TIF-1γ determination, namely, D-Tek Myositis 12 IgG Dot for BlueDiver (Diagnostic Technology, Belrose, NSW, Australia), Euroimmun Euroline Autoimmune Inflammatory Myopathies (Euroimmun, Lübeck, Germany) and Trinity Biotech ImmcoStripe Myositis Advanced LIA (Buffalo, New York, USA). However, although concordance studies exists for the three assays (11) and for D-Tek and Euroline with IP (12–15), there are no reports of the usefulness of those assays in diagnosing CAM in a large cohort of patients.

We analyzed agreement in anti-TIF-1γ antibody detection for the Euroline profile compared with our in-house IB assay with human recombinant TIF-1γ which, in a previous study, showed very good kappa agreement with IP (8). We also analyzed prevalence of anti-TIF-1γ antibodies as detected by the two methods in a cohort of DM patients and the usefulness of the methods in diagnosing CAM.

## Patients and Methods

To analyze agreement between the methods, we reviewed all Euroline and in-house IB results from 2014 to 2020 held by the Immunology Department of Hospital de la Santa Creu i Sant Pau (Barcelona, Spain). We included 266 adult patients, and the diagnosis was categorized as follows: DM (21 patients), undifferentiated connective tissue diseases (UCTDs) (127 patients), and other diseases (118 patients). The UCTD group was composed mostly of patients with Raynaud phenomenon, dry eyes or mouth or idiopathic inflammatory myopathies other than DM. The group categorized as other diseases included mostly patients with muscular involvement and/or hyperCKemia.

To analyze the prevalence of anti-TIF-1γ antibodies as detected by the two methods in a cohort of DM patients and the usefulness of the methods in diagnosing CAM, we included an additional 42 patients with DM (total sample 308 patients) from Vall Hebrón Hospital (Barcelona, Spain).

Definitive DM diagnosis was established according to Bohan and Peter criteria (16, 17) or a score of >90% according to the International Myositis Classification Criteria (18). Patients with DM were further categorized as having CAM if, 3 years before or after the diagnosis of DM, they were diagnosed with a subjacent tumor.

### Anti-TIF-1γ Determination

Euroline Autoimmune Inflammatory Myopathies 16 Ag (IgG) Profile (Euroimmun, Lübeck, Germany) was performed semi-automatically using EUROBlotMaster from Euroimmun following manufacturer instructions. Immunoblots were graded as negative or positive by at least two independent experienced observers on the basis of signal intensity.

The in-house IB with human recombinant TIF-1γ was performed as follows. Purified recombinant protein (4 µg) encoding the longest isoform of TIF-1γ (OriGene, Rockville, Maryland, USA) was run on 4-12% polyacrylamide Criterion™ Tris-Glycine Extended (TGX) Stain-Free™ precast gels for PAGE (Bio-Rad, Munich, Germany) with Tris-Glycine-SDS running buffer using the Bio-Rad criterion electrophoresis system. Western blotting was performed using the Bio-Rad trans-blot turbo transfer system on a nitrocellulose membrane. TIF-1γ-transferred nitrocellulose was incubated for 1h at room temperature (RT), in phosphate buffered saline containing 3% non-fat dry milk (blocking buffer) and maintained at -20°C until use. For testing, nitrocellulose was vertically cut into strips and incubated with human serum samples diluted 1:100 in blocking buffer for 1 h at RT with shaking. After washing, phosphatase alkaline-labeled goat anti-human IgG antibody (Invitrogen, USA), at 1:1000 dilution in blocking buffer, was added to each strip and incubated 1h at RT with shaking. Color development was with phosphatase reagent (BCIP/NBT, Sigma-Aldrich, St Louis, Missouri, USA). Positive and negative controls were included in all batches. Immunoblots were graded, by at least two independent experienced observers, as negative or positive based on signal intensity at 155 kDa molecular weight.

Determinations of anti-TIF-1γ were made as routine testing at the time of request. Samples were tested by the two methods at the same time point, avoiding freezing cycles. Results of each assay type were interpreted without knowledge of the results of the other assay.

### Statistical Analysis

Cohen’s kappa coefficient was used to measure agreement between the Euroline and in-house IB results. Fisher’s exact test was used to compare sensitivity, specificity and positive and negative predictive values (PPV and NPV, respectively) for the tests. The Delong et al. (1988) method was used to calculate the standard error for the area under the receiver operating characteristic (ROC) curve (AUC) and the difference between two AUCs. All tests were two-sided, and probability values of p<0.05 were considered statistically significant. Analyses were performed using SPSS V.21.0 for Cohen’s kappa coefficient, Graphpad Prism 7 for Fisher’s exact test, and MedCalc statistical software for the Delong et al. method.

## Results

### Anti-TIF-1γ Detection by Euroline vs In-House IB

A total of 266 patients were included in this part of the study. Agreement between Euroline and in-house IB assays is summarized in **Table 1**. The two methods agreed in 90.6% (241/266) of cases, yielding fair agreement according to Cohen’s kappa [k=0.3163; 95% confidence interval (CI): 0.1199-0.5126, p<0.0001].

**Table 1:** Anti-TIF-1γ antibody results for Euroline and in-house immunoblot.

Patients/sample	Euroline	In-house immunoblot
234/266	Negative	Negative
20/266	Positive	Negative
5/266	Negative	Positive
7/266	Positive	Positive

### Diagnostic Classification of Anti-TIF-1γ Positive Patients by Euroline vs In-House IB

Of the 308 patients included for this part of the study, 127 had UCTDs, 118 had other diseases and 63 had DM. Euroline and in-house IB assay results according to diagnosis are summarized in **Table 2**.

**Table 2:** Anti-TIF-1γ antibody detection by Euroline and in-house immunoblot by diagnostic group.

Diagnosis (patients/sample)	Euroline	In-house immunoblot	p
**DM (63/308)**	16 (25.4%)	22 (34.9%)	0.3318
**UCTDs (127/308)**	3 (2.4%)	6 (4.7%)	0.4999
**Other (118/308)**	17 (14.4%)	1 (0.8%)	0.0001

### Relationship Between CAM and Anti-TIF-1γ Detected by Euroline vs In-House IB

Of the 63 patients diagnosed with DM, 16 had CAM, of whom 8 (50%) and 12 (75%) had anti-TIF-1γ antibodies detected by Euroline and in-house IB, respectively. There were 4 patients who had anti-TIF-1γ antibodies detected by the in-house IB but not by Euroline. Of the 47 patients without CAM, 8 (17%) and 10 (21%) had anti-TIF-1γ antibodies detected by Euroline, and in-house IB, respectively.

**Figure 1:**
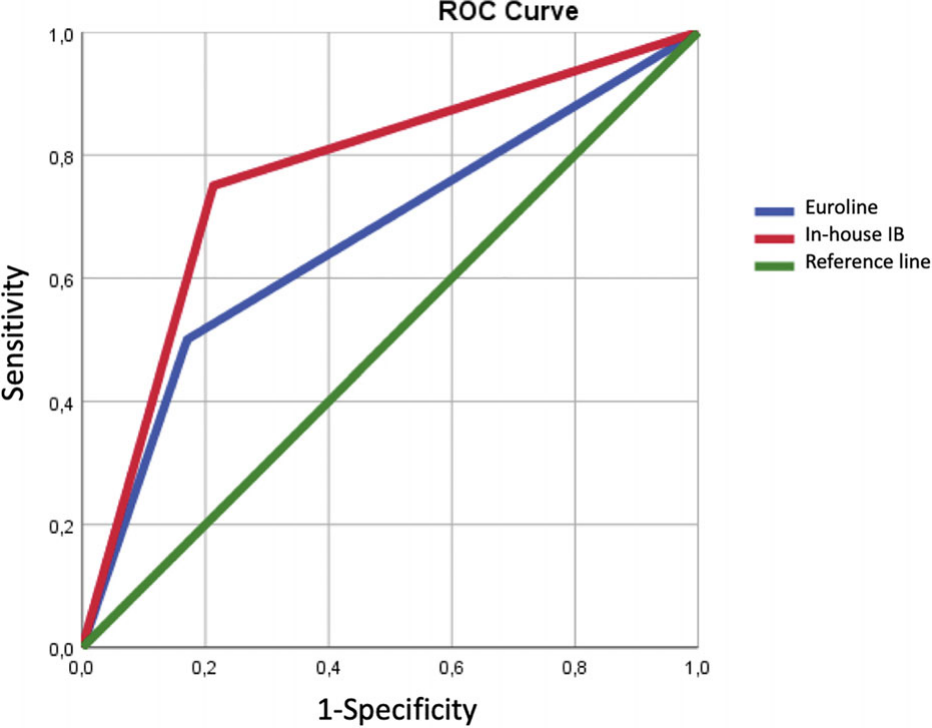
Receiver operating characteristic (ROC) curve.

In diagnosing CAM, Euroline sensitivity and specificity values were 50% and 83%, respectively, and in-house IB sensitivity and specificity values were 75% and 79%, respectively. The AUC values were 0.665 (95% CI: 0.5–0.83) for Euroline and 0.769 (95% CI: 0.628–0.91) for the in-house IB (**Figure 1**). The in-house IB compared to Euroline more reliably detected cancer in patients with DM with anti-TIF-1γ antibodies (p=0.0014 vs p=0.0502 for in-house IB vs Euroline).

**Table 3** summarizes sensitivity and specificity values, PPVs, and NPVs for CAM diagnosed based on anti-TIF-1γ antibody detection by Euroline and the in-house IB.

**Table 3:** Sensitivity, specificity and positive and negative predictive values (PPV and NPV, respectively) for anti-TIF-1γ antibody detection of cancer in patients with cancer-associated dermatomyositis.

	Euroline	In-house IB	p
**Sensitivity (%)**	50	75	0.2734
**Specificity (%)**	83	79	0.7939
**PPV (%)**	50	54.5	>0.9999
**NPV (%)**	83	90	0.3675

## Discussion

We report results for a comparison between a commercial anti-TIF-1γ assay (Euroline) and an in-house anti-TIF-1γ assay (in-house IB)—previously validated using a gold standard method, namely IP (8)—based on determining sensitivity, specificity, PPV, and NPV for a large cohort of patients.

Agreement between the Euroline and the in-house IB methods was, on the whole, fair (k=0.3163; 95% CI: 0.1199–0.5126, p<0.0001). The main disagreement was in relation to 20 anti-TIF-1γ positive results detected by Euroline but not by the in-house IB, with 16 (80%) of those results for patients in the other-diseases group. While agreement of k=0.7 between Euroline and IP has previously been established for anti-TIF-1γ antibody detection in two studies (13, 14), those studies referred to patients with myositis, while in our study the main disagreement reflected other diseases.

The usefulness of anti-TIF-1γ antibodies as a cancer biomarker has been conclusively proven only for patients with DM. However, immunology and clinical analysis laboratories receive myositis antibody (anti-TIF-1γ antibodies included) screening requests with no definitive diagnosis of DM. Other authors have found unacceptably high false positive rates (FPRs) for Euroline-detected myositis-specific and myositis-associated antibodies in healthy controls (19), so the high FPRs may to some extent be inherent to the technique. Unexpectedly high percentages of anti-TIF-1γ antibodies detected by Euroline in patients without DM dilutes the usefulness of anti-TIF-1γ as a myositis-specific antibody (1, 2, 6, 20, 21) and so reinforces the clinician’s role in correct DM diagnosis. This means that pre-test probability in any given patient is an important aspect of using Euroline for anti-TIF1γ detection. If DM diagnosis was right, there was a good agreement between Euroline and the in-house IB, agreement was poor in the other scenarios.

The presence of anti-TIF-1γ antibodies in DM by IP ranges from 13% to 33% (1, 2, 6, 20). Both Euroline and the in-house IB assays have equivalent sensitivity values that also concur with previously described anti-TIF-1γ antibody detection rates in the DM context.

Of the 16 patients with CAM in our study, four had anti-TIF-1γ antibodies detected by the in-house IB but not by Euroline (50% of false negative results). A similar false negative rate has been reported for Euroline comparisons with IP (12). The non-detection of anti-TIF-1γ antibodies potentially results in less rigorous malignancy screening and later detection and treatment.

Previous studies have reported anti-TIF-1γ antibody frequency by IP of 50%–71.4% for patients with CAM and of 4.5%–14.3% for patients without CAM (2, 6); those rates are quite similar to those obtained by both methods in our study.

In diagnosing CAM, for anti-TIF-1γ antibodies detected by IP, sensitivity and specificity have been reported as 50%–100% and 79%–100%, respectively, while pooled estimated sensitivity and specificity values in a meta-analysis were 78% and 89%, respectively (7); the sensitivity and specificity values in our study for both methods therefore lie within the ranges defined. Although the in-house IB was more accurate in diagnosing CAM than Euroline, both methods were less accurate than IP (the gold standard), as the meta-analysis reported a pooled calculated AUC for anti-TIF-1γ detected by IP of 0.91 (95% CI: 0.88–0.93) (7).

The PPV and NPV for anti-TIF-1γ antibodies detected by IP to predict CAM have been established: PPV ranged from 58% to 66.7%, while NPV ranged from 92% to 95% (6, 7). Both methods compared in this study have slightly lower PPV and NPV than those established for anti-TIF-1γ antibodies detected by IP. However, it would have been advisable to establish PPV and NPV in our cohort by IP.

Our data indicate that anti-TIF-1γ antibodies as detected by Euroline and the in-house IB have sensibility and specificity values, PPVs and NPVs similar to those reported in the literature for CAM diagnosis. However, the high number of patients positive for anti-TIF-1γ antibodies according to Euroline in the context of other diseases could mislead the initial diagnosis towards DM and, outside the context of a DM, could lead clinicians to look for cancer. No studies exist to date that associate anti-TIF-1γ antibody with cancer outside a DM diagnosis. Therefore, the high proportion of positive results in patients without DM could lead to cancer screening of patients for whom it is not known whether they are at greater risk of cancer. A limitation of this study is the relatively small representation of anti-TIF-1γ positive results.

While multiple testing increases efficiency, it also has the disadvantage of increasing the likelihood of false positive results; therefore, if anti-TIF-1γ antibodies are detected by Euroline, we strongly recommend confirming the results using a second validated method, most especially in the absence of a definitive DM diagnosis. We also recommend, to avoid false-negative results, confirming anti-TIF-1γ antibodies using a second validated method if a DM diagnosis is definitive, the Euroline result is negative for anti-TIF-1γ antibodies and other myositis-specific antibodies.

## Conclusion

Although differences in results between a commercial anti-TIF1γ assay (Euroline) and an in-house IB previously validated against IP are not significant for patients with DM and CAM, agreement in the absence of a definitive DM diagnosis is poor. Our results underpin the importance of a reliable DM diagnosis by the clinician (pre-test probability) and suggest that the usefulness of anti-TIF1γ antibody detection depends not only on the method used but also on an accurate DM diagnosis.

## Data Availability Statement

The raw data supporting the conclusions of this article will be made available by the authors, without undue reservation.

## Ethics Statement

The studies involving human participants were reviewed and approved by Institut de Recerca de l’Hospital de la Santa Creu I Sant Pau (IIB Sant Pau). Written informed consent for participation was not required for this study in accordance with the national legislation and the institutional requirements.

## Author Contributions

AM and MAM established the study design. AS-O’C, IC, MM, AG, ET-A, MA-C, and HC contributed with diagnoses of patients. AB, MS, LV-G, LA, and TF-L contributed with anti-TIF-1γ antibody detection. AM, CJ, and LM-M contributed to data analysis and manuscript writing. All authors contributed to the article and approved the submitted version.

## Conflict of Interest

The authors declare that the research was conducted in the absence of any commercial or financial relationships that could be construed as a potential conflict of interest.
